# Impact of Emotion on Consciousness: Positive Stimuli Enhance Conscious Reportability

**DOI:** 10.1371/journal.pone.0018686

**Published:** 2011-04-11

**Authors:** Kristine Rømer Thomsen, Hans C. Lou, Morten Joensson, Jonathan A. Hyam, Peter Holland, Christine E. Parsons, Katherine S. Young, Arne Møller, Alan Stein, Alex L. Green, Morten L. Kringelbach, Tipu Z. Aziz

**Affiliations:** 1 Department of Psychiatry, University of Oxford, Oxford, United Kingdom; 2 Center of Functionally Integrative Neuroscience, Aarhus University, Aarhus, Denmark; 3 Department of Neurosurgery, John Radcliffe Hospital, Oxford, United Kingdom; French National Centre for Scientific Research, France

## Abstract

Emotion and reward have been proposed to be closely linked to conscious experience, but empirical data are lacking. The anterior cingulate cortex (ACC) plays a central role in the hedonic dimension of conscious experience; thus potentially a key region in interactions between emotion and consciousness. Here we tested the impact of emotion on conscious experience, and directly investigated the role of the ACC. We used a masked paradigm that measures conscious reportability in terms of subjective confidence and objective accuracy in identifying the briefly presented stimulus in a forced-choice test. By manipulating the emotional valence (positive, neutral, negative) and the presentation time (16 ms, 32 ms, 80 ms) we measured the impact of these variables on conscious and subliminal (i.e. below threshold) processing. First, we tested normal participants using face and word stimuli. Results showed that participants were more confident and accurate when consciously seeing happy versus sad/neutral faces and words. When stimuli were presented subliminally, we found no effect of emotion. To investigate the neural basis of this impact of emotion, we recorded local field potentials (LFPs) directly in the ACC in a chronic pain patient. Behavioural findings were replicated: the patient was more confident and accurate when (consciously) seeing happy versus sad faces, while no effect was seen in subliminal trials. Mirroring behavioural findings, we found significant differences in the LFPs after around 500 ms (lasting 30 ms) in conscious trials between happy and sad faces, while no effect was found in subliminal trials. We thus demonstrate a striking impact of emotion on conscious experience, with positive emotional stimuli enhancing conscious reportability. In line with previous studies, the data indicate a key role of the ACC, but goes beyond earlier work by providing the first direct evidence of interaction between emotion and conscious experience in the human ACC.

## Introduction

Is conscious experience affected by what we feel? It has been argued that emotion and reward are intimately linked to conscious experience [1]. Specifically, evidence from human neuroimaging has shown that the same brain structures are implicated in both emotional processing and the regulation of level of consciousness [1], [2]. The anterior cingulate cortex (ACC) is pivotal in the integration of reward, attention and conscious experience [3]–[9]. More specifically, the ACC has been shown to play a crucial role in the hedonic, or affective, dimension of consciousness, e.g. as evidenced by a specific encoding of pain unpleasantness in the ACC [6]. Accordingly, the ACC could play a central role in the possible interactions between emotion and conscious experience.

Signal detection theory offers a potential route to investigate the link between consciousness and emotion, and their underlying brain mechanisms. Using this approach, conscious experience is determined by the setting of a criterion for when the sensory signal to noise ratio warrants confidence that the stimulus is present [10]. A valid measure of conscious experience is thus participants' ability to report or detect stimuli, and importantly, investigations should include both subjective and objective measures of conscious reportability [11], [12]. Previous studies of emotion generally show enhanced processing of positive compared to neutral and/or negative stimuli [13]–[15], or enhanced processing of emotional (positive and negative) compared to neutral stimuli - in terms of faster reaction time or increased accuracy [16], [17]. However, studies do not include the subjective dimension of conscious experience, in terms of participants' acknowledged perceptual awareness. In addition, most studies only focus on conscious processing, and rarely include all three types of emotion (positive, neutral and negative) separately. Overall, there is a tendency to focus on how processing of negative stimuli differs from processing of neutral, (e.g. recent studies by Gaillard et al. and Luo et al. [18], [19]).

No previous studies have tested the impact of emotion on conscious and subliminal (i.e. below threshold) processing, using objective and subjective measures of reportability. Neither has the role of the ACC in this process been tested directly.

**Figure 1:**
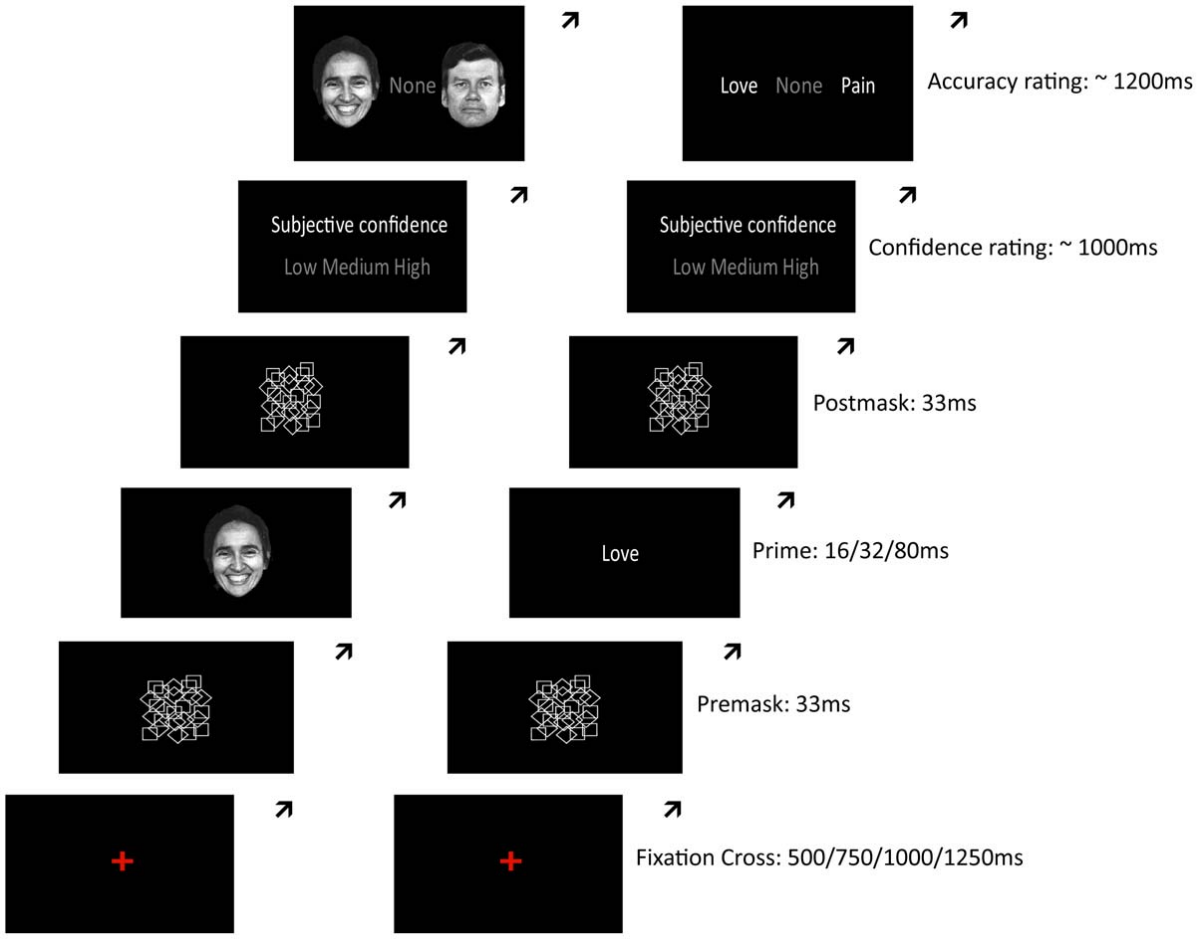
Behavioural paradigms. Faces (or words) of different emotional valence are embedded within a premask and a postmask followed by a confidence rating (low, medium, high) and a forced choice test (target, none, distracter). To ensure that participants do not respond automatically (i.e. without attending to the stimuli), the duration of the fixation cross varies, and a blank screen was added between the postmask and the confidence rating with varying duration.

To test if there is an impact of emotion on conscious experience we used a masking paradigm similar to that used by Dehaene et al. [20], but in contrast to Dehaene et al., we used both subjective confidence and objective accuracy measures of conscious reportability. In this paradigm, a briefly presented stimulus is embedded in two masks, after which participants are asked to indicate: 1) “How confident are you that you saw a stimulus”, and 2) “Which stimulus do you think you saw” (forced choice task with two distracters). In order to measure the impact of emotion on conscious and subliminal processing we varied the duration of the stimuli (16/32/80 ms; i.e. reflecting subliminal and conscious processing) and the emotion of the stimuli (positive, neutral and negative faces or words) (see **Figure 1**) (see also **Materials and Methods**).

First, a group of normal participants (N = 30) performed the task using pictures of emotionally valenced faces presented for 32 ms and 80 ms. In this young cohort both the 32 ms and the 80 ms conditions were consciously perceived (i.e. participants were above chance when identifying the faces in the forced choice task). Hence, a second study was conducted using a shorter presentation time of 16 ms, which was subliminal in this cohort (i.e. they were at chance when identifying faces in the forced choice task) (N = 15). To ensure findings could be replicated with a different type of stimuli, a third study was conducted, using positive, neutral and negative words presented visually for 16 ms and 80 ms (N = 15, all native English speakers).

The opportunity to test behavioural findings directly through electro-physiological recordings arose because we had access to a patient who had undergone successful deep brain stimulation surgery for treatment-resistant whole-body chronic pain, who had two electrodes inserted bilaterally into the ACC; close to the posterior rostral cingulate zone [21]. This provided a unique opportunity to record directly from the ACC and nearby white matter tracts while the electrodes were externalized for a week following the initial operation (see **Materials and Methods**). During performance of the task (using happy, neutral and sad faces) we recorded local field potentials (LFPs) from the ACC of the patient.

## Results and Discussion

**Figure 2:**
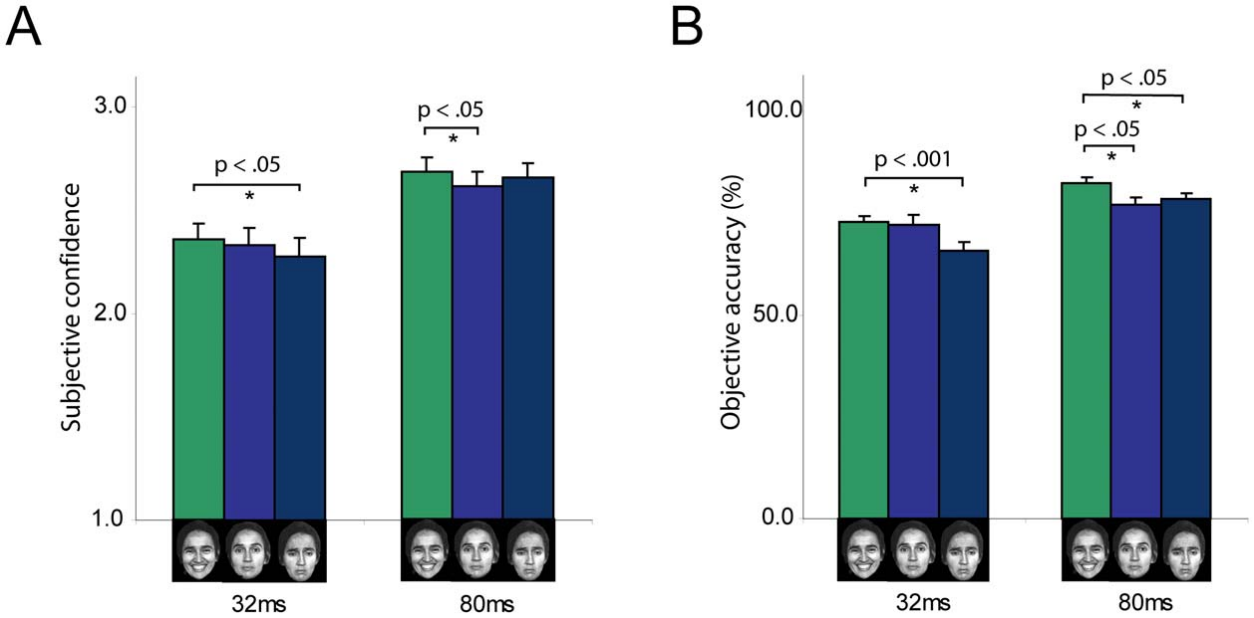
Happy compared to neutral and negative faces enhance conscious reportability measures (confidence and accuracy). When faces are presented for 32 ms, participants are more confident of seeing happy versus sad faces, and more accurate in identifying happy versus sad faces. Similarly, when faces are presented for 80 ms, participants are more confident of seeing happy versus neutral faces, and more accurate in identifying happy versus neutral faces and also happy versus sad faces. In this young cohort the 32 ms and the 80 ms conditions were both consciously perceived (i.e. participants are above chance when identifying the faces). N = 30. Error bars indicate standard error of the mean.

**Figure 3:**
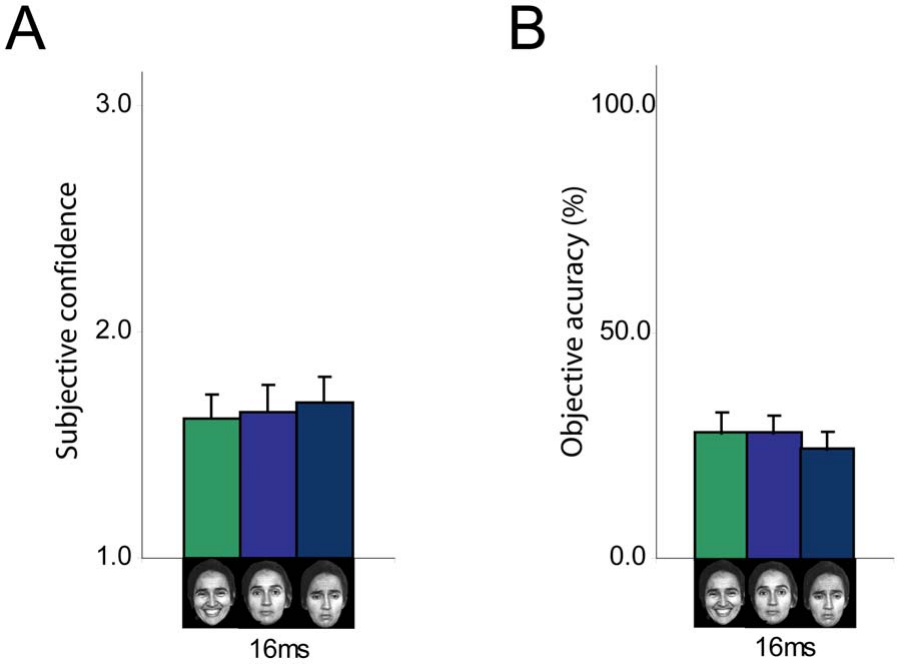
No effect of different emotional stimuli on confidence or accuracy when stimuli are presented subliminally. When faces are presented for 16 ms, which is subliminal in this cohort (i.e. participants are at chance when identifying the faces), we do not see an effect of emotion in terms of confidence and accuracy. N = 15. Error bars indicate standard error of the mean.

Results showed that in the 80 ms condition, participants were more confident of seeing happy (Mean ± Standard error; 2.69±0.07) versus neutral faces (2.62±0.07; p = 0.002), and more accurate in identifying happy (82.50±1.32) versus neutral (77.18±1.83; p = 0.029) and versus sad faces (78.68±1.26; p = 0.012). Similarly in the 32 ms condition, participants were more confident of seeing happy (2.36±0.08) versus sad faces (2.28±0.09; p = 0.005) and more accurate in identifying happy (72.75±1.61) versus sad faces (66.00±1.86; p<0.0001) (see **Figure 2**). In this cohort, the 80 ms and 32 ms conditions were both consciously perceived (i.e. participants were above chance when identifying the faces). When faces were presented for 16 ms, which was subliminal (i.e. participants were at chance when identifying the faces) we did not see an effect of emotion in terms of confidence and accuracy (see **Figure 3**).

**Figure 4:**
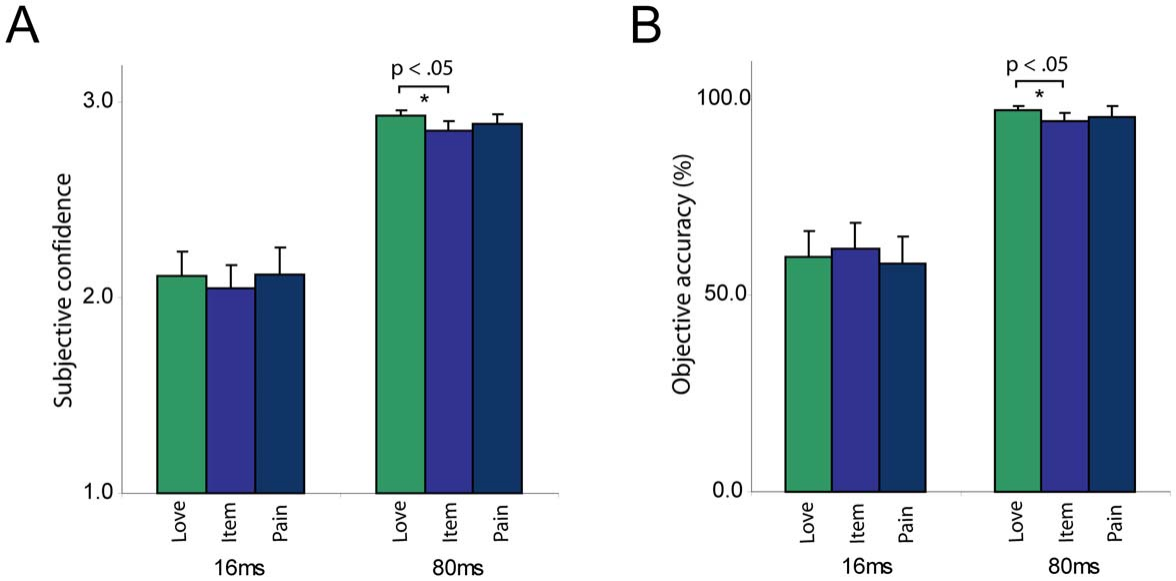
Positive compared to neutral words enhance conscious reportability. When words are presented for 80 ms, participants are more confident of seeing positive versus neutral words, and more accurate in identifying positive versus neutral words. When words are presented for 16 ms, we do not see an effect of emotion on confidence and accuracy. N = 15, all native English speakers. Error bars indicate standard error of the mean.

This effect of emotion on conscious reportability was not specific to emotional faces but also held for emotional words: In the 80 ms condition, participants were more confident of seeing positive (2.93±0.03) versus neutral words (2.86±0.05; p = 0.008), and also more accurate in identifying positive (97.00±1.72) versus neutral words (94.33±2.00; p = 0.008). When words were presented for 16 ms we did not see an effect of emotion on confidence and accuracy (see **Figure 4**).

**Figure 5:**
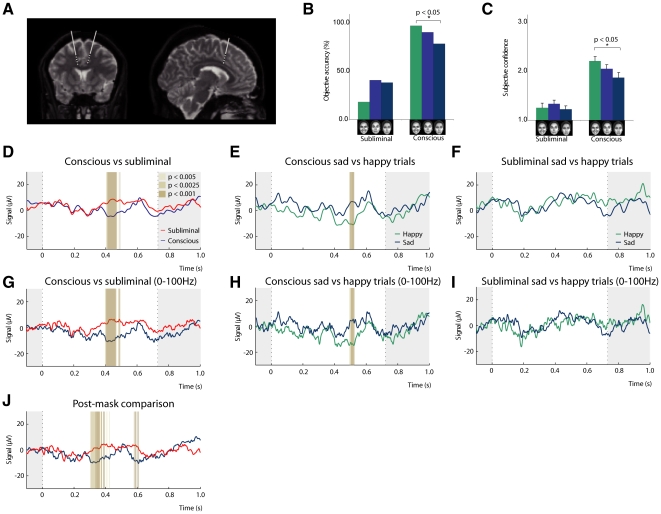
A region of the ACC shows significant differences mirroring behavioural effects with regards to conscious reportability. (**A**) Magnetic resonance imaging showing the location of the bilateral electrodes in the patient's brain normalised to MNI space with four electrode points (in black) in the ACC and nearby white matter. (**B, C**) The behavioural results in the patient showed that he was more confident of seeing faces in conscious (83 ms) versus subliminal (33 ms) trials, and more accurate in identifying faces in conscious versus subliminal trials. In the conscious trials, the patient was more confident of seeing happy versus sad faces, and also more accurate in identifying happy versus sad faces. In the subliminal trials we found no effect of emotion on conscious reportability. (**D**) Mirroring the behavioural findings, we found significant differences in the local field potentials between the two deepest electrodes in the ACC after 400 ms (lasting around 70 ms) when comparing conscious (83 ms) and subliminal (33 ms) trials. (**E**) Similarly, in the conscious trials, we found significant differences slightly later after 496 ms (lasting 30 ms) between happy and sad faces. (**F**) Importantly, this significant effect of emotion was not found in the subliminal trials. (**G–I**) When we applied a lowpass-filter with a 100 Hz cut of frequency the comparisons yielded the same results as in **D–F** where a 30 Hz cut of frequency was used (see Methods). (**J**) To rule out potential effects on LFP activity by the difference in stimulus presentation time in subliminal and conscious trials, we reanalysed this comparison linked to end of postmask, rather than start of the stimuli, and found the same significant difference in LFP activity between conscious and subliminal trials.

In our deep brain stimulation patient, an initial control task showed that stimuli presented for 33 ms was subliminal (i.e. he was at chance when identifying the faces) while stimuli presented for 83 ms was consciously perceived (i.e. he was above chance when identifying the faces). Hence, the task was conducted using happy, neutral and sad faces shown at 33 ms and 83 ms. For ethical reasons we only used one type of stimuli, to reduce the risk of painful sensations in the patient, following lack of stimulation during recordings from the ACC (see **Figure 5A**).

Results showed that he was more confident of seeing faces in conscious (2.1±0.05) versus subliminal trials (1.28±0.05; p<0.0001), and more accurate in indentifying faces in conscious (Mean 87.78) versus subliminal trials (Mean 32.22; p<0.0001). In line with results from the normal participants, the patient was more confident of seeing happy (2.21±0.09) versus sad faces (1.87±0.10; p = 0.014) in the conscious trials, and also more accurate when identifying happy (Mean: 96.97) versus sad faces (Mean: 78.57; p = 0.025) in the conscious trials. Again, we found no effect of emotion on conscious reportability when faces were presented subliminally (see **Figure 5B and 5C**). Together with behavioural findings from the first cohort, this provides evidence for the important impact of emotion on conscious reportability, and thus on conscious experience.

Importantly, behavioural findings were mirrored in our recordings from the ACC: we found significant differences in the LFPs between the two deepest electrodes after 400 ms (lasting around 70 ms) when comparing conscious and subliminal trials (p<0.001) (see **Figure 5D and 5G**). Similarly, in the conscious trials, we found significant differences slightly later after 496 ms (lasting 30 ms) between trials of happy and sad faces (p<0.001) (see **Figure 5E and 5H**). Crucially, in the subliminal trials, we did not see an effect of emotion in the neural activity, which is consistent with our behavioural findings (see **Figure 5F and 5I**).

We thus demonstrate a striking impact of emotion on conscious experience, with positive emotional stimuli enhancing conscious reportability, which is central to the regulation of consciousness. Previous studies have shown a similar effect [13], [14], [15], but to our knowledge, this is the first study to show an impact of positive stimuli on conscious experience across different types of stimuli (faces as well as words) and measures (objective as well as subjective).

Our findings highlight the need to include positive stimuli in future investigations of interactions between emotion and conscious experience, possibly by looking into the role of dopamine in reward. We recently demonstrated that dopaminergic stimulation can enhance confidence and accuracy when seeing rapidly presented words [22]. Dopaminergic neurotransmission is also likely to play an important role in the enhanced processing of positive compared to negative and neutral stimuli reported here, given its well-documented role in the motivational component of reward processing, ‘wanting’ or incentive salience [23]. Therefore dopaminergic neurotransmission is integrated in both emotion and conscious experience.

Our finding that LFP activity is part of the neural processing of both emotion and conscious experience is interesting and also challenging. The brain processing of any stimuli requires complex and intricate communication between multiple regions. The ACC is near the top of the hierarchy of brain processing. The combined effects of emotion and conscious experience on LFPs in the ACC may be a culmination of a complex chain of brain events, including a possible role of attention and working memory. In order to rule out potential effects on LFP activity by the difference in stimulus presentation time for conscious and subliminal stimuli, we reanalysed the LFP data linked to the end of the postmask rather than the start of the stimuli. This comparison still yielded a significant difference in the LFP activity between conscious and subliminal activity (see **Figure 5J**).

Overall, the data indicate a key role of the ACC in the reported interaction. This is in line with previous findings showing involvement of the ACC in the affective component of conscious experience, but goes beyond earlier work by providing the first direct evidence of interaction between emotion and consciousness in the human ACC.

Together, our findings suggest an intimate relationship between consciousness and emotion. Future research should help to clarify the possible role of dopaminergic reward in the reported impact of positive stimuli on conscious experience, and help to further characterize the spatiotemporal components of a larger whole-brain network. The latter was recently done using a similar paradigm, but with only negative and neutral faces [19], thereby precluding further insight into the interaction between positive stimuli and conscious experience reported here.

## Materials and Methods

### Ethics Statement

This study was given a favourable ethical opinion by Oxfordshire REC B on 17 June 2008.

### Experimental Design and Procedures: Behavioural Studies

The behavioural studies were carried out in 2009 at University Department of Psychiatry, Oxford, UK. The studies were approved by Oxfordshire Research Ethics Committee. Participation was voluntary, and written informed consent was obtained prior to participation. Each experimental session lasted approximately 25 minutes, and participants were tested alone to ensure anonymity. Participants were recruited through poster advertisement at Colleges and Laboratories in Oxford. None of the participants had previously participated in a similar experiment. Participants were included if they did not suffer from any neurological, psychiatric or psychological problems and did not receive medication affecting the brain.

In total, 30 participants were included in the first behavioural study, investigating possible effects of emotion on conscious and subliminal processing (using face stimuli presented for 32 ms and 80 ms). However, in this young cohort the 32 ms condition was not subliminal (i.e. they were above chance when identifying the faces). In addition, we wished to ensure findings were not related to differences in facial features, e.g. seeing white teeth in happy faces could facilitate improved recognition. Hence, 15 of the participants (all native English speakers) were tested in two additional studies: (a) looking at performance in a subliminal condition (using faces, presented at 16 ms), and (b) looking at performance using word stimuli, presented at 16 ms and 80 ms.

#### Behavioural Task

The behavioural paradigm was based on the assumption that both subjective and objective measures are needed in the conceptual understanding and experimental study of consciousness [11], [12]. The behavioural paradigm was designed to measure conscious reportability using subjective (i.e. confidence) and objective (i.e. accuracy in a forced choice task) measures. In the task, the participant is presented with a red fixation cross followed by a face (16/32/80 ms), which is embedded in two masks of 33 ms duration [24]. Following this, the participant is asked to indicate: (a) subjective confidence of having seen a face (*low*, *medium*, or *high*), and (b) which face he thinks he saw from three forced choices (*target*, *distractor*, or *no face*). To ensure that participants do not respond automatically (i.e. without attending to the stimuli), the duration of the fixation cross varied (500/750/1000/1250 ms), and a blank screen was added between the postmask and the confidence rating (600/850/1100/1350 ms) (see **Figure 1**). Due to the refresh rate of the screen, there was a blank gap of up to 16.5 ms duration between onset of images. The task consisted of three types of trials: (1) faces presented for 32 ms, (2) faces presented for 80 ms, and (3) blank trials (80 ms).

The task was programmed in Presentation software (Version 14.4, Neurobehavioural Systems, Inc. www.neurobs.com). Participants performed the task on a Samsung NC10 laptop computer (screen width: 800, screen height: 600, screen depth: 32, refresh rate: 62.5 Hz).

#### Face stimuli

The face stimuli used included both infants and adults with three types of emotion: happy, neutral and sad. Faces are important stimuli that help us direct and adapt our behaviour to our surroundings, and since the emotional valence is easily recognized, faces are suitable for investigations of emotions. The total number of trials in the task was 270, consisting of 120 subliminal trials (32 ms), 120 conscious trials (80 ms) and 30 blank trials (80 ms). Trials were randomised in the task, and all faces were shown an equal amount of times (including as distractor faces).

The infant faces used in the task are from a database of digital photographs of infant facial expressions that was produced from digital videotapes of 27 infants (ages 3–12 months) [25]. They were filmed in their own homes with approval from Oxfordshire Research Ethics Committee. The database is unique in that it contains pictures of the same infants expressing positive, neutral and negative emotions, controlled for head direction and eye gaze, and in that they are all matched for attractiveness, emotional expression and arousal. The adult faces are from the Ekman database [26], and have been matched with the infant faces on valence, arousal, attractiveness, luminosity and size.

#### Word stimuli

To ensure findings could be replicated with a different type of stimuli, a sub-group of the participants (all native English speakers) performed the same task, but with positive, neutral and negative words. The word stimuli was selected from “The Affective Norms for English Words” (ANEW [27]) which contains over 1000 English words rated on valence, arousal and dominance (see **Table 1**). In addition, the words were matched to ensure that the three groups (positive, neutral and negative words) were not statistically different from each other in terms of imagine-ability (p = 0.950), frequency (p = 0.992), number of letters (p = 0.982), number of phonemes (p = 0.921) and number of syllables (p = 0.932), but statistically different in terms of ratings of valence (p = 0.0001) (see **Table 2**).

**Table 1:** Word Stimuli.

Word Type	Positive		Neutral		Negative	
	Baby	Love	Avenue	Locker	Abuse	Hell
	Beach	Lucky	Basket	Month	Afraid	Hurt
	Cash	Orgasm	Black	Pencil	Alone	Injury
	Cheer	Proud	Bowl	Phase	Anger	Jail
	Comedy	Puppy	Column	Poster	Bomb	Killer
	Cute	Peace	Cord	Sour	Cancer	Misery
	Flirt	Reward	Fabric	Sphere	Corpse	Pain
	Friend	Sexy	Finger	Stiff	Crash	Poison
	Gift	Spring	Foot	Spray	Cruel	Prison
	Happy	Sunset	Hard	Table	Death	Rape
	Honest	Talent	Item	Taxi	Dead	Slave
	Honor	Thrill	Knot	Theory	Devil	Stupid
	Joke	Wise	Lamp	Tower	Hate	Victim

Positive, neutral and negative words from Affective norms for English words (ANEW).

**Table 2:** Matching of Positive, Neutral and Negative Words.

Word type	IMG	KFREQ	NLET	NPHN	NSYL	VAL	ARSL
Positive	521.11	54.32	5.04	4.25	1.52	7.93	5.96
Neutral	524.14	55.38	5.04	4.13	1.58	5.10	3.88
Negative	516.52	53.12	5.08	4.21	1.58	2.02	6.07

Mean values. IMG, Imagineability; KFREQ, Frequency; NLET, number of letters; NPHN, number of phonemes; NSYL, number of syllables; VAL, rating of valence from 1 (very negative) through 5 (neutral) to very positive (8); ARSL, rating of arousal from very low (1) through medium (5) to very high (9).

#### Descriptive

30 participants (15 female, 15 male) were included in the first behavioural study, mean age 27.3 years (Standard error ±5.7). Out of these, 15 (9 male, 6 female) also participated in the two following studies: (a) looking at performance in a subliminal condition, and (b) using a different type of stimuli, words, mean age 26.8 years (±5.9).

### Experimental Design and Procedures: Direct investigation of the ACC

The electrophysiological study was carried out in a chronic pain patient having undergone deep brain stimulation (DBS) surgery in 2009 at the John Radcliffe Hospital, Oxford, UK. The study was approved by Oxfordshire Ethics Committee. Participation was voluntary, and written informed consent was obtained prior to participation. In addition, the patient has given written informed consent to publication of his case details. The experimental session lasted approximately 25 minutes, and the patient had not previously participated in a similar experiment.

The 49-year old patient underwent DBS surgery after struggling with chronic whole-body pain for over 10 years from high cervical injury following severe motorbike accidents. The target structure to treat his chronic pain was the posterior part of the anterior cingulate cortex (pACC); close to the posterior rostral cingulate zone [21].

#### Surgical procedures

A T2-weighted MRI scan of the patient's brain was performed prior to surgery [28]. For surgery, a Cosman-Roberts-Wells Stereotactic frame was fixed to the patient's head under local anesthesia. A computed tomography (CT) scan was then performed and using the Radionics Image Fusion® and Stereoplan® (Integra Radionics, Burlington, Mass) program the coordinates for the target in pACC were calculated. The target was set on axial image 25 mm posterior to the tip of the frontal horn of the lateral ventricle. After a Stereotactic frame guided twist drill craniostomy, a Radionics™ electrode was passed to 5 mm from target, measuring impedance during its course. The DBS electrode (Medtronic 3387® electrode (Medtronic Inc., Minneapolis, USA)) was then placed to target, on each side. Clinical testing was performed and the electrode was fixed to the skull, and externalised.

The whole stimulation system was then internalised one week later in a second procedure under general anaesthesia, where extension leads were used to connect the electrodes to the implantable pulse generator (IPG – kinetra™, Medtronic). The laboratory-based experiment was performed in the week between these two procedures.

#### Local Field Potential Recordings

The experiment took place in a quiet room, kept at a constant 22 degrees Celsius. The patient sat in a comfortable chair in a semi-recumbent position. Local field potentials (LFPs) were simultaneously recorded with bipolar configuration from the adjacent four circumferential contacts of each DBS macroelectrode. Signals were filtered at 0.5–500 Hz and amplified (×10,000) using isolated CED 1902 amplifiers and digitised using CED 1401 Mark II at a rate of 2480 Hz (Cambridge Electronic Design, Cambridge, UK). LFPs were then displayed online and saved onto hard disk using Spike II software® (version 5.0, Cambridge Electronic Design).

#### Behavioural Task

The patient was tested using the same paradigm (see **Figure 1**), but with a total of 240 trials: 105 trials with faces presented for 33 ms, 105 trials with faces presented for 83 ms and 30 blank trials (33 ms). The number of trials was reduced for ethical reasons, to reduce the risk of painful sensations in the patient, following lack of stimulation during recordings. Prior to the experimental session, a brief control task was administered to ensure that the presentation times reflected a subliminal and a conscious condition. The optimal timings for the patient were 33 ms and 83 ms for the subliminal and conscious condition respectively.

The patient performed the task on a computer running Windows XP Pro (Microsoft, Seattle, USA), equipped with a parallel output cable that delivered triggers to the LFP recording system at the onset of each visual stimulus and button press with millisecond precision. The experiment was shown on a TFT screen using a screen resolution of 800×600×32 and a refresh rate of 60 Hz.

### Analysis and Statistical Details

The experiments included two types of behavioural measures: 1) subjective ratings of confidence (1: low, 2: medium, 3: high) and 2) objective measures of accuracy (percentage of correct responses).

SPSS software was used to analyse the behavioural data (Version 16, SPSS Inc., www.spss.com). Since the data was not normally distributed, non-parametric tests were applied. Wilcoxon was used to test for differences between trials of different emotion in the behavioural studies. In the analysis of the patient the three emotion conditions (happy, neutral and sad) were analysed as independent samples. Hence, Mann-Whitney was used to test for differences between trials of different emotion in terms of confidence, and Pearson Chi-Square was used to test for differences in accuracy (correct or incorrect). We used the Quartile or Fourth-Spread method to remove outliers.

MATLAB 7.9.0.529 was used to analyse the electrophysiological data recorded from the pACC in the patient undergoing DBS surgery. The data was filtered both with a 5^th^ order Butterworth lowpass-filter with a 30 and a 100 Hz cut of frequency and 2^nd^ order Butterworth notch filter to remove line power noise (50 Hz). The trials were baseline corrected based on a 100 ms pre-stimuli window and trials with excessive variance were excluded from the analysis. A trial average was calculated for each condition with each trial fixated to stimuli onset (i.e. presentation of faces). A rank sum analysis (Wilcoxon rank-sum test for equal medians) was used with confidence intervals of 0.1%, 0.25% and 0.5%. The earliest onset of next event is illustrated on the figures to ensure effects from this are not misinterpreted.
